# Metabolomics in Early Alzheimer's Disease: Identification of Altered Plasma Sphingolipidome Using Shotgun Lipidomics

**DOI:** 10.1371/journal.pone.0021643

**Published:** 2011-07-11

**Authors:** Xianlin Han, Steve Rozen, Stephen H. Boyle, Caroline Hellegers, Hua Cheng, James R. Burke, Kathleen A. Welsh-Bohmer, P. Murali Doraiswamy, Rima Kaddurah-Daouk

**Affiliations:** 1 Sanford-Burnham Medical Research Institute, Orlando, Florida, United States of America; 2 Department of Medicine, Duke-NUS Graduate Medical School, Singapore, Singapore; 3 Department of Psychiatry and Behavioral Sciences, Duke University Medical Center, Durham, North Carolina, United States of America; 4 Bryan Alzheimer Disease Research Center, Duke University Medical Center, Durham, North Carolina, United States of America; 5 Duke Institute of Brain Sciences, Duke University Medical Center, Durham, North Carolina, United States of America; The University of Hong Kong, Hong Kong

## Abstract

**Background:**

The development of plasma biomarkers could facilitate early detection, risk assessment and therapeutic monitoring in Alzheimer's disease (AD). Alterations in ceramides and sphingomyelins have been postulated to play a role in amyloidogensis and inflammatory stress related neuronal apoptosis; however few studies have conducted a comprehensive analysis of the sphingolipidome in AD plasma using analytical platforms with accuracy, sensitivity and reproducibility.

**Methods and Findings:**

We prospectively analyzed plasma from 26 AD patients (mean MMSE 21) and 26 cognitively normal controls in a non-targeted approach using multi-dimensional mass spectrometry-based shotgun lipidomics [1], [2] to determine the levels of over 800 molecular species of lipids. These data were then correlated with diagnosis, apolipoprotein E4 genotype and cognitive performance. Plasma levels of species of sphingolipids were significantly altered in AD. Of the 33 sphingomyelin species tested, 8 molecular species, particularly those containing long aliphatic chains such as 22 and 24 carbon atoms, were significantly lower (p<0.05) in AD compared to controls. Levels of 2 ceramide species (N16:0 and N21:0) were significantly higher in AD (p<0.05) with a similar, but weaker, trend for 5 other species. Ratios of ceramide to sphingomyelin species containing identical fatty acyl chains differed significantly between AD patients and controls. MMSE scores were correlated with altered mass levels of both N20:2 SM and OH-N25:0 ceramides (p<0.004) though lipid abnormalities were observed in mild and moderate AD. Within AD subjects, there were also genotype specific differences.

**Conclusions:**

In this prospective study, we used a sensitive multimodality platform to identify and characterize an essentially uniform but opposite pattern of disruption in sphingomyelin and ceramide mass levels in AD plasma. Given the role of brain sphingolipids in neuronal function, our findings provide new insights into the AD sphingolipidome and the potential use of metabolomic signatures as peripheral biomarkers.

## Introduction

As the world population ages, the number of older adults developing dementia is estimated to quadruple by mid-century to over 106 million worldwide [3]. Alzheimer's disease (AD) is the most common dementia among all the clinically-recognized dementias in the aging population [4], [5]. Although the cause is not known, there are profound biochemical alterations in multiple pathways in the AD brain including changes in amyloid-beta protein metabolism, tau phosphorylation, membrane lipid dysregulation and synaptic neurotransmission. Recent pathological, biochemical, and genetic studies have led to major insights into AD pathogenesis [6]. Among the encouraging observations are links made between cardiovascular risk conditions, lipid metabolism, and the development of AD [7]. Further, the apolipoprotein E4 (ApoE4) genotype, a major determinant of risk for sporadic AD, is a component of lipoproteins in plasma and brain and may lie at the crossroads between the lipidome and dementia [8]. These insights suggest that treatments aimed at reducing risk conditions may hold promise to delay the onset of AD dementia, slow the progression, and possibly prevent AD altogether. Since disease modifying drugs are likely to be most effective early in the disease course, the discovery of highly sensitive biomarker(s) for the early diagnosis of AD (at mild or even preclinical stages) is extremely important. Currently, the sensitivity and specificity of the clinical diagnosis of AD is quite low (approximately 80%; [9]) and the currently available central AD biomarkers (MRI volumetrics, FDG-PET, CSF assays) perform at similar accuracy levels [10], [11]. Moreover, effective biomarkers are also very useful in assessing disease progression or response to treatment. The cellular lipidome, consisting of hundreds of thousands of lipid species, represents a rich source of biomarkers for the early diagnosis of AD.

Metabolomics tools enable us to study the metabolome, the repertoire of small molecules present in cells and tissue [12], [13], [14]. The identities, concentrations, and fluxes of these substances are the final product of interactions between gene expression, protein expression, and the cellular environment. Metabolomics tools have been used to characterize metabolic signatures for several diseases including depression [15], [16], [17], motor neuron disease [18], Parkinson's disease [19], cocaine and opiate addiction [20], [21], schizophrenia [22], [23], [24], and Alzheimer's Disease [25]. As stated previously, there is now extensive preclinical and in-vitro literature documenting a relationship between altered lipid metabolism and amyloidogenesis, oxidative stress and apoptosis [26]. Previous studies with post-mortem brain tissue samples have demonstrated altered lipidomes at the different stage of AD pathogenesis (reviewed in [27]. For example, multiple classes of sphingolipids are altered not only at the late stage of the disease [28], but also at the earliest clinically recognizable stage of AD [29], [30]. All major classes of phospholipids are ubiquitously decreased at the late stage of AD [31]. Among these, the levels of plasmalogen (a major component in nerve tissue membranes counting for up to 85% of ethanolamine glycerophospholipid, or ∼30% of total phospholipids of these membranes) are gradually reduced as progress of AD severity [32]. It is conceivable that these changes could lead to or be parallel with the changes of either the content or the composition or both of the lipid species in plasma of AD patients. There are many obvious advantages to a peripheral plasma marker (over central markers) in terms of cost, risks and ability to monitor individuals over time. A very recent study has shown a promising relationship between altered plasma ceramide levels and hippocampal volume loss in mild cognitive impairment supporting the hypothesis that disruptions in ceramide metabolism may be linked to early neurodegenerative and pathologic changes [33]. However, the lack of sensitive and efficient “mega” lipid biochemistry platforms has limited the ability to simultaneously study the dozens of different pathways that may be affected in AD in relation to clinical phenotype and genotype.

Shotgun lipidomics studies the pathways and networks of cellular lipids in biological systems on a large scale. It involves the identification and quantitation of hundreds of cellular lipid molecular species as well as their interactions with other species. Multi-dimensional mass spectrometry-based shotgun lipidomics (MDMS-SL) is one of the leading analytic platforms in current lipidomics practices due to its high accuracy, efficiency, sensitivity, and reproducibility [1], [2]. In this study, we used a non-targeted MDMS-SL approach to measure the levels of over 800 molecular species of choline glycerophospholipid (PC), lysoPC (LPC), ethanolamine glycerophospholipid (PE), phosphatidylinositol, sphingomyelin (SM), ceramide, and triacylglycerol (TAG) in the plasma of AD individuals and cognitively normal controls. The main goals of the study were to examine the hypothesis that the plasma lipidome is altered in early AD, and secondary goals were to characterize lipid changes that might be differentially related to cognitive status or genotype as well as to identify potential lipid pathways that might be disrupted in AD.

## Materials and Methods

### Materials

Synthetic phospholipids including 1,2-dimyristoleoyl-*sn*-glycero-3-phosphocholine (14:1-14:1 PC), 1,2-dipalmitoleoyl-*sn*-glycero-3-phosphoethanolamine (16:1-16:1 PE), 1,2-dipentadecanoyl-*sn*-glycero-3-phosphoglycerol (15:0-15:0 PG), 1-myristoyl-2-hydroxyl-*sn*-glycero-3-phosphocholine (14:0 LPC), N-lauroyl sphingomyelin (N12:0 SM), and N-heptadecanoyl ceramide (N17:0 ceramide) were purchased from Avanti Polar Lipids, Inc. (Alabaster, AL, USA). Note that the prefix “N” denote the amide linkage of the fatty acyl chain. Triheptadecenoylglycerol (T17:1 TAG) was purchased from Nu-Chek Prep, Inc. (Elysian, MN, USA). Deuterated cholesterol was purchased from Cambridge Isotope Laboratories, Inc. (Cambridge, MA, USA). All the solvents were obtained from Burdick and Jackson (Honeywell International Inc., Burdick and Jackson, Muskegon, MI, USA). All other chemicals were purchased from Sigma-Aldrich (St. Louis, MO, USA).

### Subjects

AD (n = 26) and elderly control subjects (n = 26) were recruited prospectively from the Joseph and Kathleen Bryan Alzheimer's Disease Research Center (Bryan ADRC) and the Department of Psychiatry, both at Duke University. Patients included in the study had diagnoses of Probable AD (NINDS) using current criteria following history and informant interview, complete neurological examination, cognitive screening and securing standard blood chemistries, imaging, and laboratory studies. The Mini-Mental State examination (MMSE), a brief screening tool of cognitive status was used to categorize AD patients as mild or moderate, consistent with routine clinical practice. Normal controls were participants who were determined to be free of dementia as well as other significant neuropsychiatric disorders after reviewing their medical history, informant interviews, functional status and neuropsychological performance as well as ratings of mood and clinical examinations. Our entry criteria excluded subjects with current major depression or substance abuse, subjects with other neurologic disorders such as Parkinson's disease or stroke, and those who were unwilling or unable to perform cognitive testing. Of the 26 AD subjects, 17 had mild AD (MMSE≥20) and others had moderate AD. The demographic and clinical characteristics of subjects appear in Table 1.

**Table 1:** Clinical and demographic characteristics of Alzheimer's subjects and controls^a^.

	Control	AD	P	Test
n	26	26		
Age Range	57–87	61–89		
Age, Mean (SD)	73.0 (7.7)	77.2 (6.5)	0.074	WT
% Male	42	46	1	FET
Years Education, Mean (SD)	16.0 (2.6)	14.4 (2.8)	0.062	WT
MMSE, Median (MAD)	29.0 (1.5)	21.0 (3.7)	2.50E-09	WT
Logical Memory I, Median (MAD)	17.0 (4.4)	2.0 (1.5)	1.00E-09	WT
Logical Memory II, Median (MAD)	15.5 (3.7)	0.0 (0.0)	5.10E-10	WT
% Using Statins	27	42	0.38	FET
% with APOE4 Genotype	25%	67%	-	-

a WT, Wilcoxon rank-sum test, two-sided; FET, Fisher's exact test, two-sided; SD, standard deviation; MAD, median absolute deviation; MMSE, Mini-Mental State Examination.

### Ethics statement

All normal control subjects and AD patients provided informed consent prior to collection of any data. For AD patients, informed consent was also obtained from their legally authorized representative/family member if deemed appropriate. This research was approved by the Duke University Medical Center institutional review board and was conducted according to the principles expressed in the Declaration of Helsinki.

### Lipid sample preparation from human plasma

Fasting samples were collected and cold stored using a standardized procedure. After fasting overnight, a 10 mL K3EDTA tube of blood was collected via venipuncture. The blood was mixed by gentle inversion 8–10 times and placed on ice until centrifuged for 30 minutes at 3500 rpm's at 4°C. After centrifuging, plasma was aliquoted and then frozen at −80°C. All samples were run blinded. A protein assay on each plasma sample was performed by using BCA method with bovine albumin as standard. After 200 µl of plasma from each plasma sample was transferred to a disposable culture borosilicate glass tube (16×100 mm), a premixed lipid solution used as internal standards for quantification of lipid species was added to each plasma sample based on its protein concentration. These internal standards include 15:0-15:0 PG (150 pmol/mg protein), 16:1-16:1 PE (15 pmol/mg protein), 14:1-14:1 PC (2000 pmol/mg protein), T17:1 TAG (3000 pmol/mg protein), 14:0 LPC (1000 pmol/mg protein), N12:0 SM (500 pmol/mg protein), N17:0 ceramide (15 pmol/mg protein), and others. Addition of these internal standards allows the final quantified lipid content to be normalized to both the protein content and/or the plasma sample volume and allows the elimination of a potential loss from the incomplete recovery. These internal standards have been selected because they only represent ≪0.1% of the endogenous cellular lipid mass as demonstrated by ESI/MS lipid analysis.

Lipid extracts were prepared by using a modified procedure of Bligh and Dyer as previously described [34] and each was resuspended in 500 µl of dichloromethane/methanol (1∶1, v/v) which corresponded to a concentration of 3 nmol/µl. A portion of each individual lipid extract (approximately 100 µl) was treated with LiOMe and followed by being washed with hexane as previously described [35]. The treated lipid samples were used for the analysis of the sphingolipidome of each individual plasma sample. Another portion of each individual original lipid extract was washed with 2 ml of hexane for twice to remove most of the non-polar lipids such as TAG, cholesterol, and cholesterol esters which are very abundant in plasma lipid extracts and interfere with the quantification of phospholipid classes. The residue lipid solution after washing was used for the analysis of phospholipids by using intrasource separation followed by employing multi-dimensional mass spectrometric analysis.

### Lipidomic analysis of human plasma lipid extracts by multi-dimensional mass spectrometry-based shotgun lipidomics (MDMS-SL)

A triple-quadrupole mass spectrometer (Thermo Fisher TSQ Vantage, San Jose, CA, USA) equipped with an automated nanospray apparatus (i.e., Nanomate HD, Advion Bioscience Ltd., Ithaca, NY) and Xcalibur system software were utilized in the study as previously described [36]. Each originally-prepared lipid extract was diluted to <50 pmol/µl with dichloromethane/methanol/isopropanol (1∶2∶4, v/v/v) prior to infusion to the mass spectrometer for the analyses of neutral lipids including TAG, cholesterol, and cholesterol esters. Each lipid solution prepared after treatment with LiOMe or after washing with hexane was also properly diluted prior to infusion to the mass spectrometer for the analyses of sphingolipids or phospholipids, respectively. Proper dilution of the lipid solution is crucial for quantification to guarantee no lipid aggregation is formed during analysis and minimizing any effects of residual inorganic components carried over during lipid extraction on ion suppression and/or chemical noise. The diluted lipid extract was directly infused through the nanomate device. Typically, a 1-min period of signal averaging in the profile mode was employed for each survey scan. For tandem mass spectrometry, a collision gas pressure was set at 1.0 mTorr but the collision energy was varied with the classes of lipids as described previously [2]. Typically, a 2 to 5-min period of signal averaging in the profile mode was employed for acquisition of each tandem MS spectrum. All the MS spectra and tandem MS spectra were automatically acquired by a customized sequence subroutine operated under Xcalibur software.

Mass spectra in survey scanning mode were acquired after intrasource separation of each prepared and properly diluted lipid solution as previously described [37], [38]. TAG species were identified and quantified directly from the originally-prepared lipid solution as described in detail [39]. Cholesterol and cholesterol esters were quantified directly from the originally-prepared lipid solution as previously described [40]. Ceramide and SM species were identified and quantified directly from lipid solutions after treatment with LiOMe or hexane washing [41], [42]. PC and LPC species were identified and quantified directly from lipid solution after washing with hexane as described [42]. PE and PI species were identified and quantified directly from lipid solution with hexane washing as previously described in detail [2], [43]). In summary, each individual lipid species corresponding to each of the ion peaks was identified using multi-dimensional mass spectrometry through building block analyses [2]. The identified species were quantified using a two-step approach as previously described [2]. First, the abundant and non-overlapping molecular species of a class shown in one of the survey scans was quantified by comparison with a pre-selected internal standard of the class after ^13^C de-isotoping. Next, some or all of the determined molecular species of the class (plus the pre-selected internal standard) were used as standards to determine the mass content of other low-abundance or overlapping molecular species using one or multiple tandem mass traces (each of which represents a specific building block of the class of interest) by two-dimensional MS.

### APOE Genotyping

APOE genotypes were determined using TaqMan-based allelic discrimination assay (Applied Biosystems). Specifically, APOE status was established using two separate genotypes: (1) rs429358 334 T/C (ABI assay ID: C_3084793_20), and (2) rs7412 472T/C (ABI assay ID: C_904973_10). Assays were conducted per manufacturer's protocol, using 10 ng of DNA from each subject per assay. Fluorescence outputs were quantified in real time using a 7900HT Fast Real Time PCR System and the data will be analyzed using SDS software v.2.2.2 (Applied Biosystems). APOE genotype assignments were grouped for analyses as E4+ or E4−. APOE genotype data was only available for 49 subjects (34 controls and 15 AD, See **Table 1**).

### Statistical Analysis

We performed statistical analyses using the R statistical programming system [44] (http://www.R-project.org). We treated the variables MMSE, Logical Memory I, and Logical Memory II as ordered categorical variables, and therefore, rather than reporting means and standard deviations, we report medians and median absolute deviations (R function ‘mad’, defined as 1.4826 times the median of the absolute value of the deviation from the median). We used two-sided Wilcoxon rank sum tests for differences in location for these variables as well as for age and years of education. We used two-sided Fisher's exact tests for independence of affection status from each of (a) the number of males, (b) the number of subjects using statins, and (c) the number of subjects with at least one *APOE* e4 allele. For tests of differences between two independent groups, we used Wilcoxon rank sum tests to compute p-values, as the underlying variables are not in general normally distributed. We calculated Q values (cumulative false discovery rates) using the R ‘qvalue’ package [45]. We used two-sided Wilcoxon signed rank tests of the null hypothesis that the distribution of differences in standardized means is symmetric around 0 (R function ‘wilcox.test’).

## Results

### Shotgun lipidomics analysis of plasma samples

**Figure 1:**
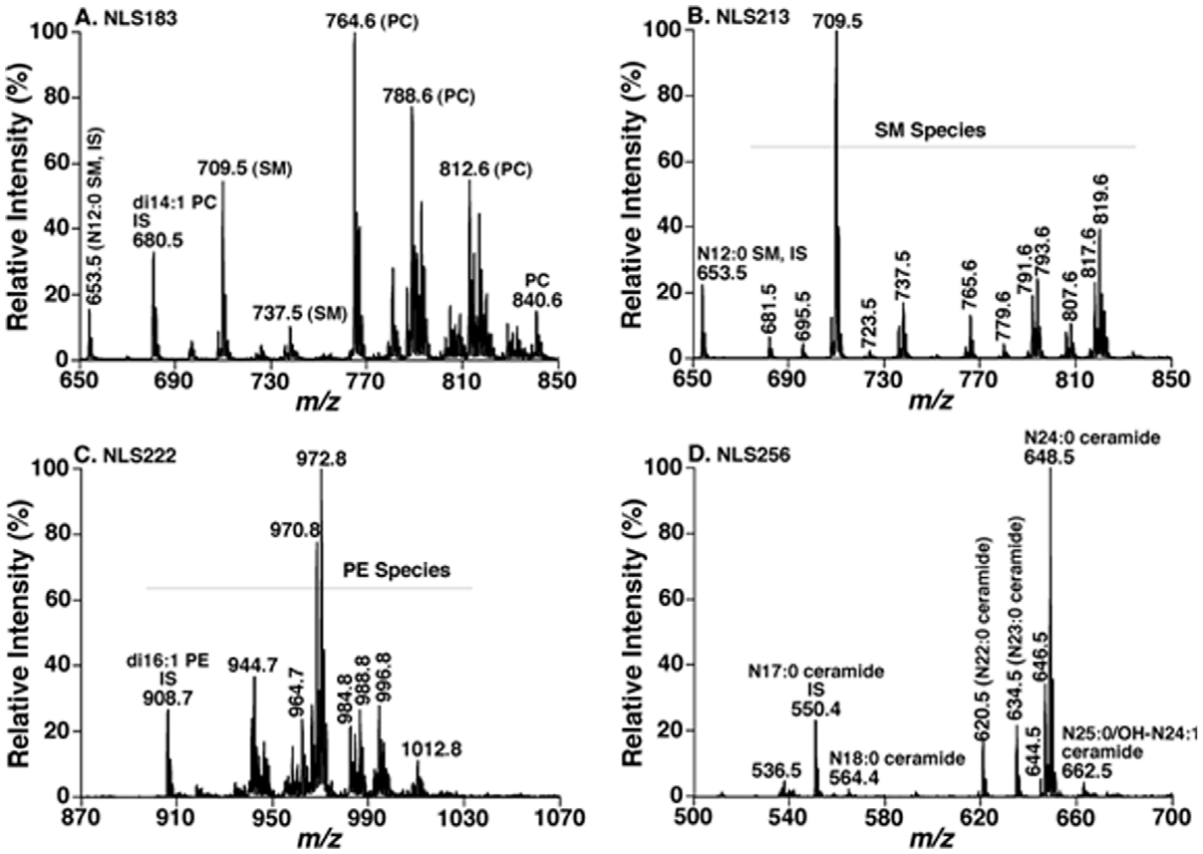
Representative tandem mass spectrometric analyses of lipid classes present in a human plasma lipid extract. Lipid samples were prepared and mass spectrometric analysis of individual sample was performed as described in “Material and Methods” in details. Panel A shows a mass spectrum of NLS183 (corresponding to the neutral loss of phosphocholine) displaying the presence of PC and SM species in the hexane-washed lipid solution; the spectrum was acquired in the positive-ion mode after addition of a small amount of LiOH to the lipid solution. Panel B shows a mass spectrum of NLS213 (corresponding to the neutral loss of phosphocholine plus methyl aldehyde) displaying the presence of SM species in the LiOMe-treated lipid solution; the spectrum was acquired in the positive-ion mode after addition of a small amount of LiOH to the lipid solution. Panel C shows a mass spectrum of NLS222 (corresponding to the neutral loss of Fmoc) displaying the presence of PE species in the hexane-washed lipid solution; the spectrum was acquired in the negative-ion mode after *in situ* addition of Fmoc-Cl to the lipid solution. Panel D shows a mass spectrum of NLS256 (corresponding to the neutral loss of 2-palmitoleyl aldehyde and a water molecule) displaying the presence of ceramide species in the LiOMe-treated lipid solution; the spectrum was acquired in the negative-ion mode after addition of a small amount of LiOH to the lipid solution. IS denotes internal standard; NLS stands for neutral loss scan.

**Figure 2:**
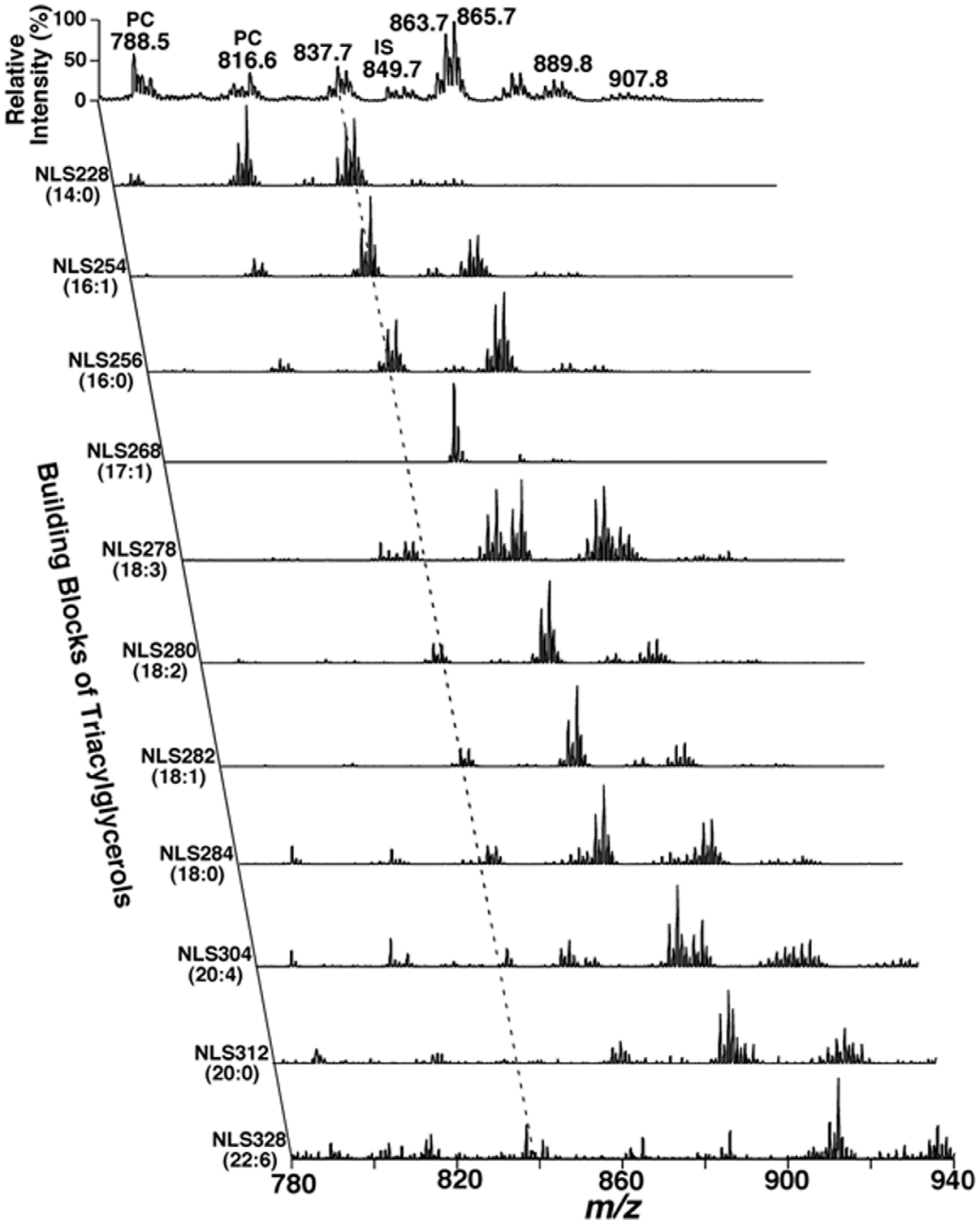
Representative 2D MS analyses of TAG species in a human plasma lipid extract. Neutral loss scans (NLS) of all naturally-occurring aliphatic chains (i.e. the building blocks of TAGs) of a human plasma lipid extract were used to determine the identities of each lithiated molecular ion, deconvolute isomeric species, and quantify individual TAG species by comparisons with a selected internal standard (i.e., T17:1 TAG, shown in NLS268). Collision activation was performed with collision energy of 32 eV and gas pressure of 1 mT on a triple quadrupole mass spectrometer (TSQ Quantum Ultra Plus, Thermo Fisher Scientific, San Jose, CA, USA). All displayed mass spectral traces are normalized to the base peak in each trace.

We analyzed 9 lipid classes in the study including choline glycerophospholipid (PC), lysoPC (LPC), ethanolamine glycerophospholipid (PE), phosphatidylinositol, sphingomyelin (SM), ceramide, triacylglycerol (TAG), cholesterol and cholesterol esters by multi-dimensional mass spectrometry-based shotgun lipidomics [1], [2]. This platform has previously employed for quantitative analysis of lipidomes (e.g., brain tissues and cerebrospinal fluid) from AD patients [29], [46]. A few representative tandem MS spectra for identification and second step quantification of PC, PE, SM, and ceramide species were shown in Figure 1. A two dimensional mass spectrum for identification and quantitation of TAG species was also illustrated (Figure 2). For example, the primary ion at *m/z* 837.7 was crossed with the 14:0, 16:0, 16:1, 18:0, 18:1, and 18:2 fatty acyl building blocks (see the broken line in Figure 2). A lithiated triacylglycerol species at *m/z* 837.7 must contain 53 total carbon atoms with 2 double bonds or 54 total carbon atoms with 9 double bonds, and the ion intensities resulting from the neutral loss of the three acyl chains from a given TAG molecule are nearly equal [39]. Thus, isomeric TAG species of 14:0-18:1-18:1, 14:0-18:0-18:2, 16:0-16:0-18:2, and 16:0-16:1-18:1 were identified. Other TAG species corresponding to other primary ions were identified similarly. The numbers of molecular species and the total masses of each individual lipid class were tabulated (Table 2). For example, we identified 65 PC molecular species present in human plasma lipid extracts with mass levels of 30±9 and 25±5 nmol/mg protein (i.e., 1890±567 and 1575±315 nmol/ml) in control and AD individuals, respectively (p = 0.022). These results are well comparable with those determined by LC-MS, which determined a total mass of 1974 nmol/ml from 31 PC species [47]. In exploratory analyses, the mass levels of total PC and SM were lower in AD patients in comparison to the control group (Table 2). Since we determined over 800 molecular species and covered the categories of (phospho)glycerolipids, sphingolipids, and sterols in the study, it is very difficult to process the dataset and make a simple comparison. Accordingly, in the current report, we mainly focused on the detailed analyses of sphingolipids at the level of individual molecular species.

**Table 2:** Lipid classes, species and their mass levels in Alzheimer and control subjects^a^.

Lipid Class	# of Species	ControlMean ± SD	ADMean ± SD	*P*
PC	65	30±9	25±5.1	*0.022*
PE	86	1.66±0.54	1.53±0.52	0.41
PI	25	0.51±0.15	0.44±0.15	0.11
SM	33	9.35±3.78	7.33±2.38	*0.026*
LPC	14	2.57±0.70	2.41±0.59	0.39
Ceramide	29	0.21±0.08	0.23±0.07	0.26
Free.Chol.	1	16±6.2	16±6	0.85
Chol.Ester	NA	32±13	32±13	0.96
TAG	>500	11.6±5.5	13.5±6.0	0.24

a SD denotes standard deviation. P values were computed by Welch two-sample t-test. All the results are obtained by shotgun lipidomics as described under Materials and Methods and presented in unit of nmol/mg protein. See text for abbreviations.

### Mass levels of sphingomyelin species in AD patients

**Figure 3:**
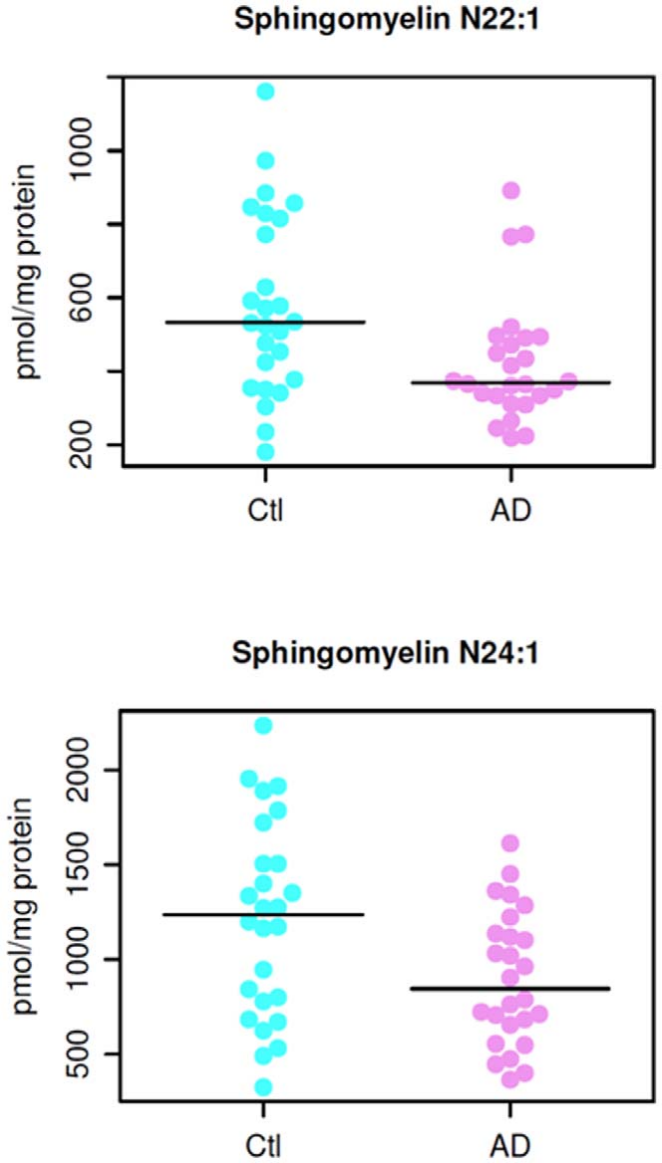
Mass levels of N22:1 (panel A) and N24:1 (panel B) sphingomyelin species in AD and healthy controls.

Wilcoxon rank sum tests revealed significant differences in the quantified sphingomyelin species between the AD and control groups. Of the 33 SM species tested, we found that 8 SM molecular species, particularly those containing long aliphatic chains such as 22 and 24 carbon atoms, were significantly lower (*p*<0.05) in AD patients as compared to the age-matched cognitively normal controls (Table 3). For example, Figure 3A displays the distribution of the mass levels of SM species N22:1 in the AD and control groups and Figure 3B shows the mass distribution of N24:1 SM species between the groups. The mass levels of additional 6 sphingomyelin species are different between AD and control groups at the *p*<0.1 level.

**Table 3:** Sphingomyelin species significantly different between AD and control groups^a^.

Sphingomyelin Species	P-value	Q-value
N22:1	0.006	0.146
N20:0	0.012	0.146
N22:0	0.014	0.146
N23:0	0.020	0.146
N23:1	0.025	0.146
N24:1	0.029	0.146
N21:0	0.034	0.146
N24:0	0.035	0.146
N17:1	0.073	0.192
N24:2	0.077	0.192
N18:0	0.084	0.192

a *as determined by Wilcoxon rank sum tests at a p-level of <0.1.*

Q values are cumulative false discovery rates estimated by the R qvalue package.

### Mass levels of ceramide species in AD patients

**Figure 4:**
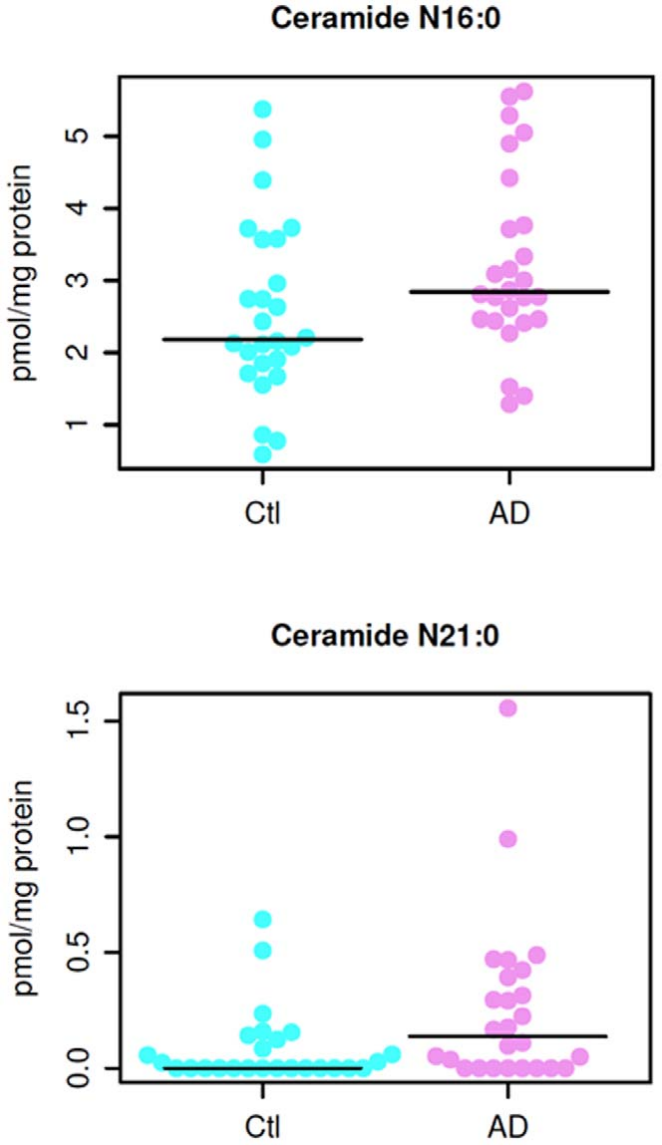
Mass levels of N16:0 (panel A) and N21:0 (panel B) ceramide species in AD and healthy controls.

In contrast to the reduction of sphingomyelin mass levels in plasma of AD patients, we identified significantly increased mass levels of 2 ceramide species (N16:0 and N21:0) (*p*<0.05) (see Figure 4 below for distribution in individual patients between the groups) and of other 5 species at the *p*<0.1 level in plasma of AD patients in comparison to those of controls (Table 4). The rest of the ceramide species were not significantly different between the AD and control groups.

**Table 4:** Ceramide species significantly different between AD and control groups^a^.

Ceramide Species	P-value	Q-value
N21:0	0.027	0.146
N16:0	0.030	0.146
N26:0	0.058	0.192
OH-N24:2	0.063	0.192
OH-N24:1	0.077	0.192
N28:2	0.078	0.192
N23:0	0.081	0.192

a *as determined by Wilcoxon rank sum tests at a p-level of <0.1.*

Q values are cumulative false discovery rates estimated by the R qvalue package.

### Ratios of ceramide to sphingomyelin species containing identical fatty acyl chain

**Figure 5:**
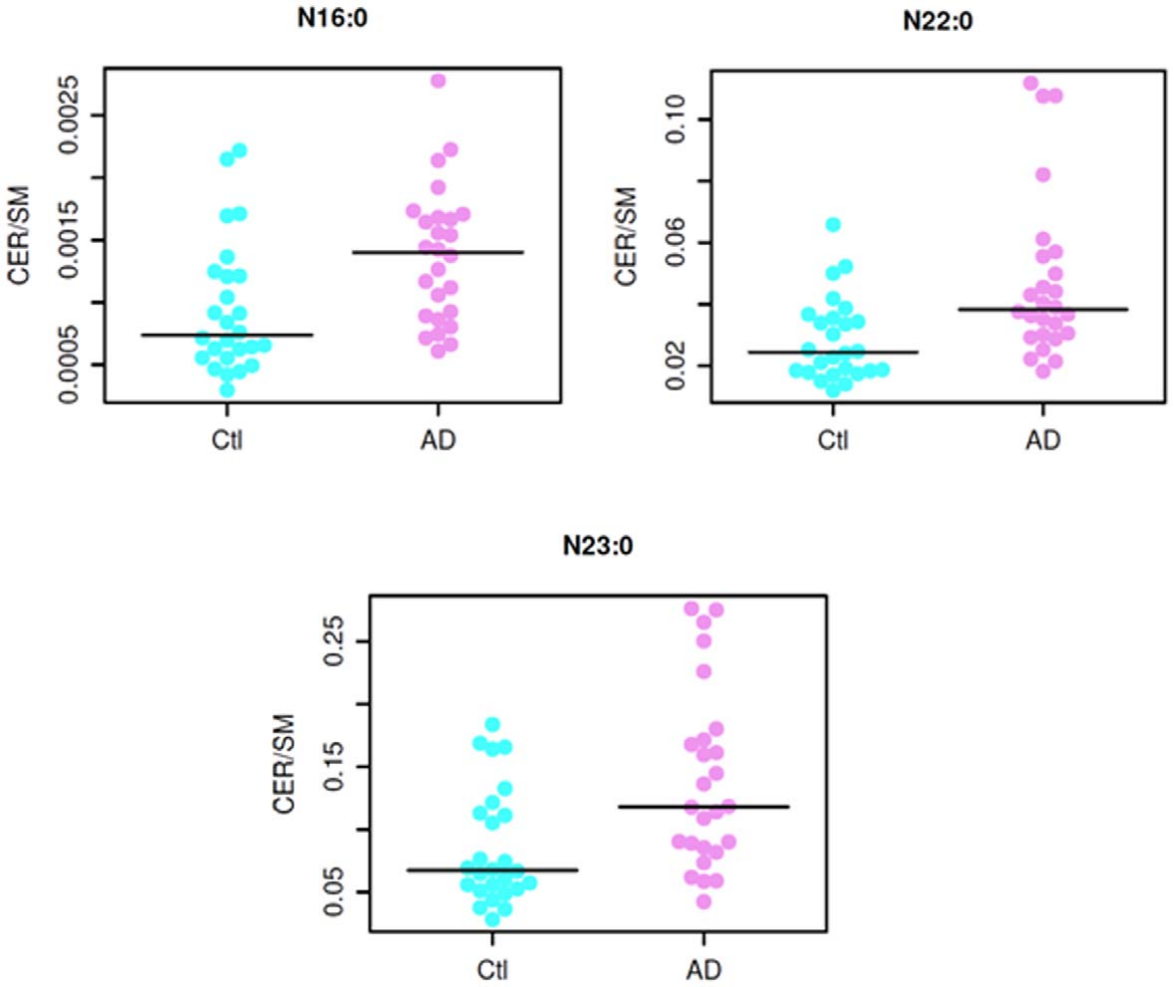
Ratios of specific ceramides to the sphingomyelins with the same fatty acid chains. See Table 5 for P values.

We computed ratios of ceramides to sphingomyelins, and then used Wilcoxon rank sum tests to compare ratios between groups. Numerous ratios differed significantly between the groups. The ratios appeared to discriminate AD versus controls more robustly than the differences seen with individual ceramide or sphingomyelin species (Table 5 and Figure 5 compared to Tables 2 and 3 and Figures 3 and 4, respectively).

**Table 5:** Ratios of ceramides to sphingomyelins with same fatty acid chain show multiple significant differences between Alzheimer's patients and controls^a^.

Fatty Acid Chain	ControlMean ± SD	ADMean ± SD	P
N22:0	0.028±0.013	0.047±0.027	0.00094*
N23:0	0.085±0.046	0.139±0.071	0.002*
N16:0	0.001±0.001	0.001±0.001	0.00214*
N21:0	0.001±0.001	0.004±0.005	0.00595
N24:1	0.027±0.013	0.042±0.026	0.00785
N24:0	0.352±0.167	0.544±0.359	0.01367
N24:2	0.003±0.002	0.005±0.005	0.0218
N18:1	0.00005±0.00015	0.00041±0.002	0.31139
N15:0	0.0001±0.001	0±0	0.33628
N22:1	0.00036±0.00049	0.001±0.001	0.45193
N23:1	0.001±0.002	0.001±0.002	0.62063
N19:0	0.005±0.019	0.001±0.002	0.63989
N22:2	0.002±0.006	0.001±0.002	0.77069

a P values were computed by Wilcoxon rank sum test, two sided.

*denotes significance at a Bonferroni corrected alpha of 0.0038.

### Sphingolipidome Alterations in Mild versus Moderate AD

**Figure 6:**
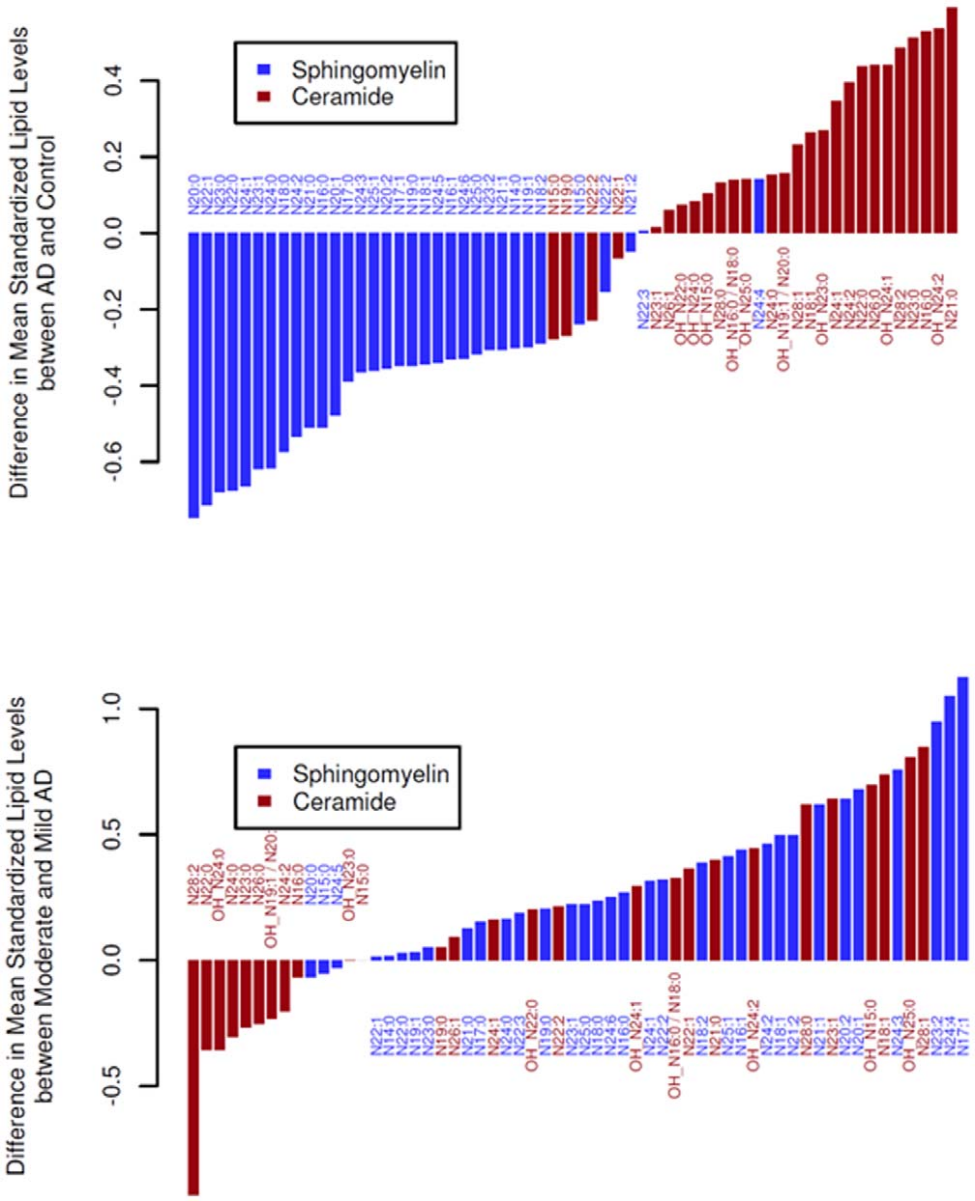
Rank of the changed mean mass levels of individual sphingomyelin and ceramide molecular species in all AD patients relative to the normal controls (panel A) and the differences in sphingomyelins and ceramides between patients with moderate Alzheimer's disease (MMSE<20) and patients with mild Alzheimer's disease (MMSE> = 20) (panel B). The Y axes show the standardized differences in lipid level: lipid levels were standardized to a mean of 0 and standard deviation of 1 across all samples before ranking and plotting. For Alzheimer's disease versus controls, P = 1.6×10^−9^ for sphingomyelins and P = 0.00034 for ceramides (Wilcoxon signed rank test two sided on means over the standardized data). For moderate versus mild Alzheimer's disease, P = 7.1×10^−8^ for sphingomyelins and P = 0.10 for ceramides.

The rank of the mean mass levels of all determined individual SM and ceramide species indicates that the reduction of SM mass levels and the increases in ceramide mass levels in AD plasma samples are essentially in uniformity, with only a few exceptions (two SM species and four ceramide species which are located at the end of small changes) (Figure 6A).

Identical data analysis was performed to compare the mass levels of these classes in mild AD patients (MMSE> = 20) and in moderate AD patients (MMSE<20) relative to the normal controls, respectively. We found that the changes of both lipid classes in either mild AD patients (p = 2.3e-10 for SM and p = 0.021 for ceramides) or moderate AD patients (p = 0.0066 for SM and p = 0.00013 for ceramides) relative to the controls are significant (Figure 6B).

**Figure 7:**
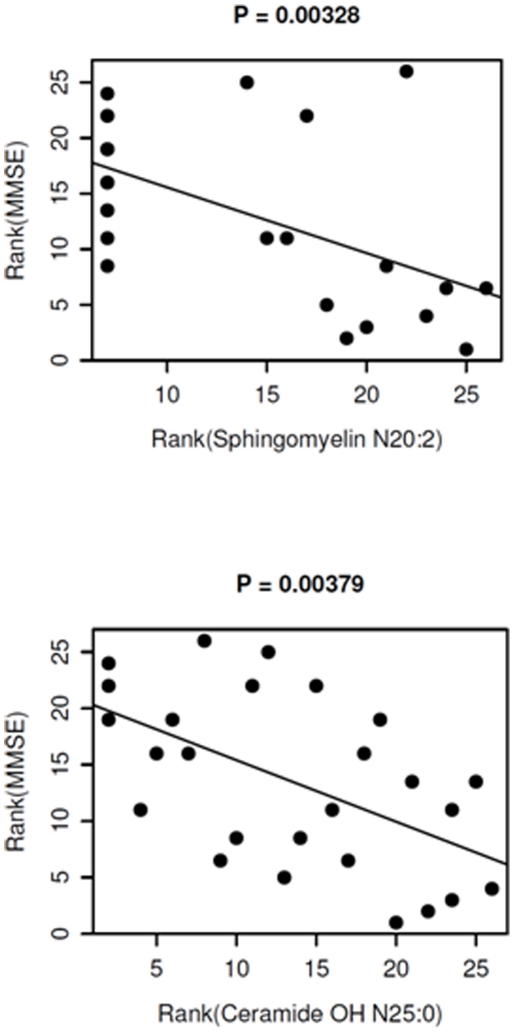
Correlation of the rank of the changed mean mass levels of sphingomyelin and ceramide species with the rank of AD severity. P values are based on Pearson's product moment correlation coefficient between the ranks as plotted. The lines represent linear regressions of the ranks of the MMSE scores against the ranks of the lipids.

Mass levels of many sphingomyelin and ceramide species were correlated with AD severity (Figure 7). For example, the rank of AD severity (i.e., MMSE) was significantly correlated with the rank of the altered mass levels of both N20:2 SM and OH-N25:0 ceramide (p values of 0.00328 and 0.00379, respectively).

### Exploratory analyses of the effects of statins on mass levels of SM and ceramides

**Figure 8:**
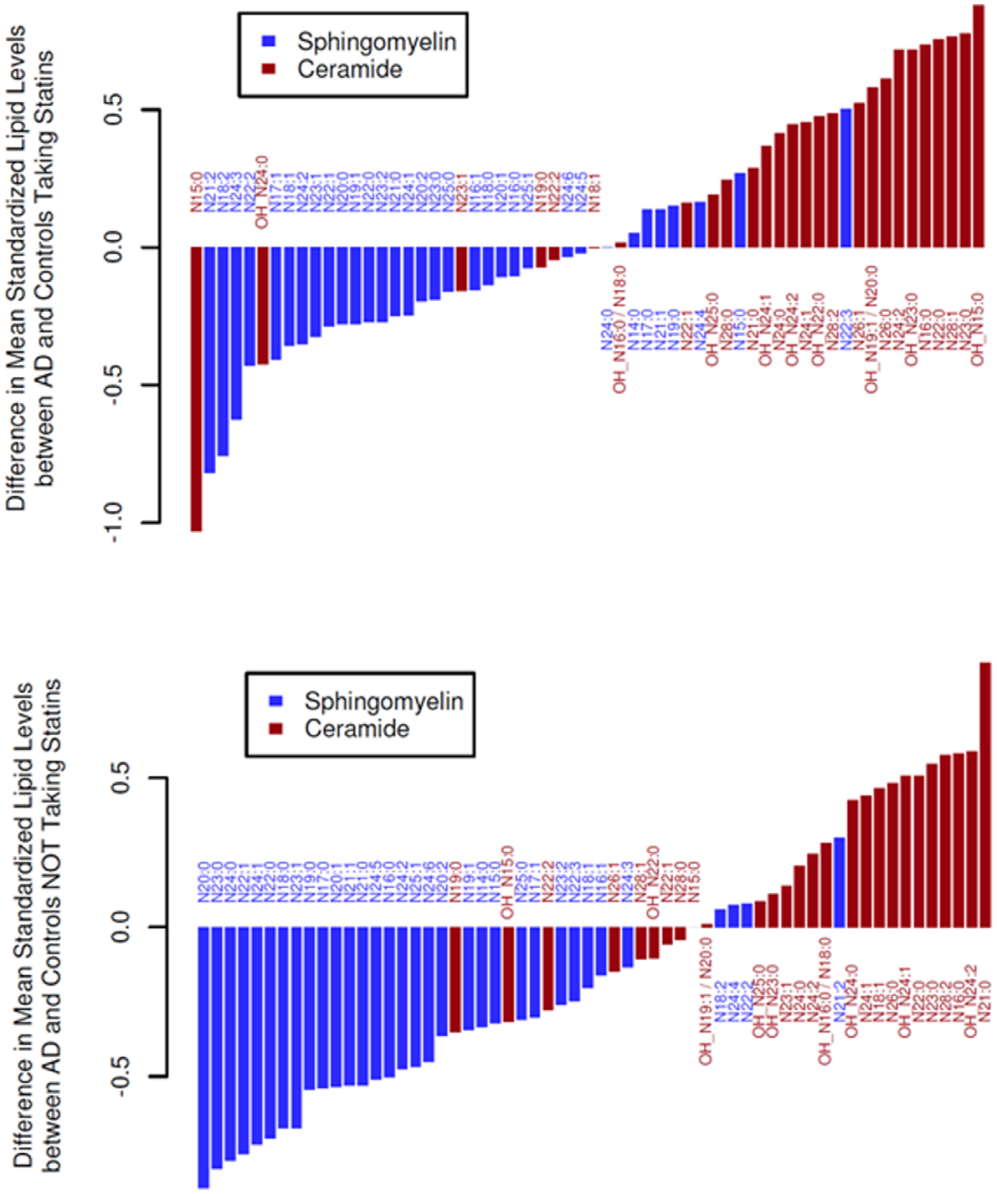
Lower sphingomyelins and higher ceramides in Alzheimer's patients are independent of statin use. Panel A shows the differences in sphingomyelin and ceramide mass levels between AD patients and controls who were taking statins. For this comparison, P = 7×10^−4^ for sphingomyelins and P = 0.00054 for ceramides (Wilcoxon signed rank test). Panel B shows the differences of sphingomyelin and ceramide mass levels between AD patients and controls who were not taking statins. For this comparison, P = 3.2×10^−8^ for sphingomyelins and P = 0.0056 for ceramides (Wilcoxon signed rank test).

Concomitant statin use is common in the elderly and may confound the identification of the altered plasma SM and ceramide profiles in AD. Our data suggests that statins significantly lowered levels of both SM and ceramides in both AD and controls (Figure 8). However, this effect does not significantly affect our main finding of lower SM mass levels and higher ceramide contents in AD patients relative to the controls (Figure 8).

### Effect of APOE4 genotype on the mass levels of plasma lipidome

**Figure 9:**
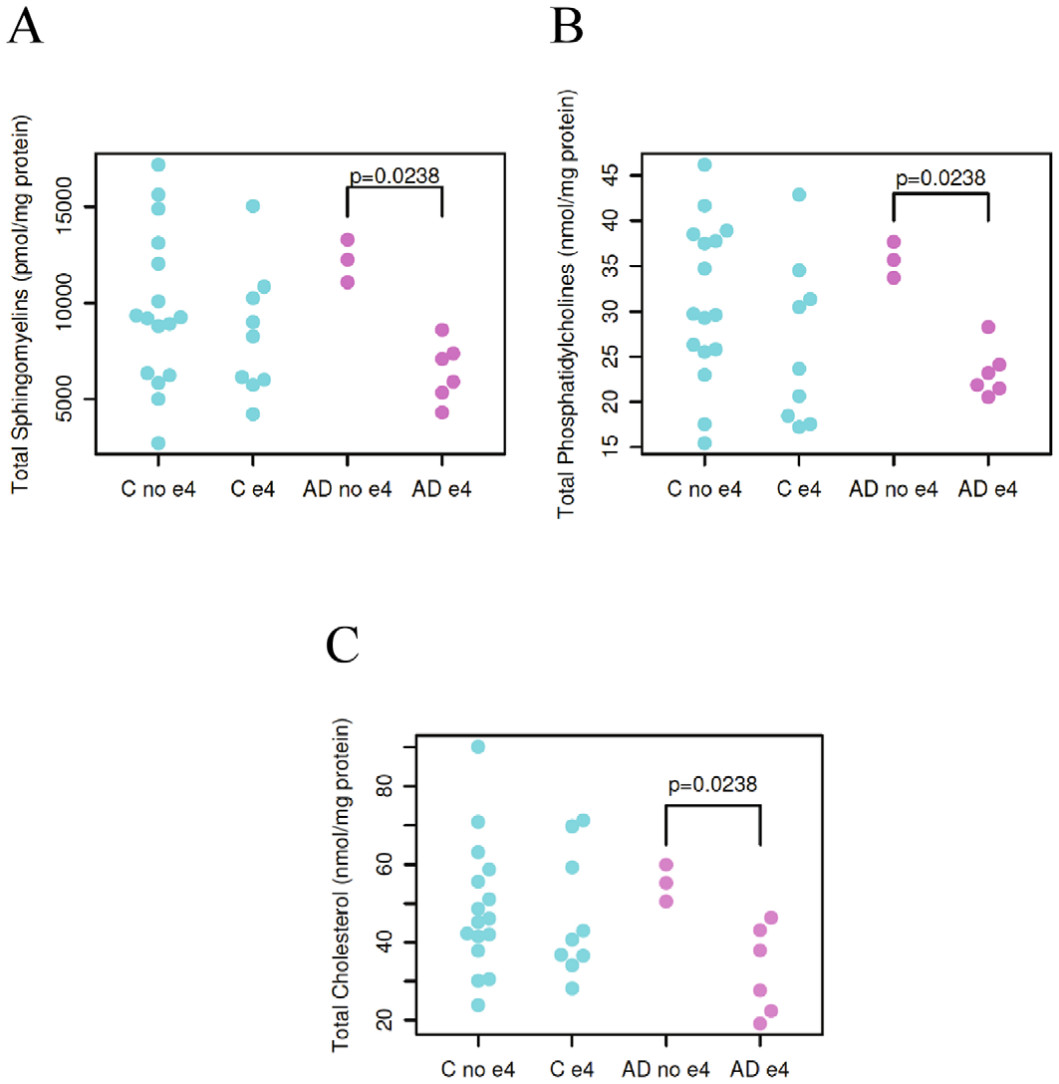
ApoE4 carriers with AD have significantly lower mass levels of sphingomyelin (panel A), phosphatidylcholine (panel B), and cholesterol (panel C) (p = 0.0238 for both lipid classes by Wilcoxon rank sum tests, two-sided).

In exploratory analyses, there was no significant relationship between *APOE* genotypes and the altered SM and ceramide profiles. However, mass levels of a few plasma lipid classes including SM, phosphatidylcholine, and cholesterol were significantly lower in AD patients carrying *APOE4* allele(s) than those carrying other *APOE* isoforms, but such mass differences were not present in normal controls (Figure 9).

## Discussion

This is the first prospective study, to our knowledge, to utilize a highly efficient multidimensional shotgun lipidomic platform to examine over 800 lipid species in plasma of a well characterized sample of AD and control subjects. The median MMSE of our AD was 21 which is in the mild range, but we also included patients with moderate AD in order to represent the range of severity seen usually in outpatient clinics. There were several key findings that emerged: First, we demonstrated significant reductions of sphingomyelin mass and significant increases in ceramide content in plasma of AD patients. Second we demonstrated that the rank order of these opposing changes are essentially species specific and uniform. Third, we showed that ratios of SM and ceramide species with identical fatty acyl chains results in more robust discrimination than either metabolite alone. Fourth, we showed that our findings are seen both in mild and moderate stages of AD with some metabolites also showing correlations with cognitive performance. In addition, although the numbers of samples genotyped are small our data suggest ApoE4 genotype specific metabolite differences in AD. The small sample size also made it difficult to account for interference from systemic metabolism and the stage of disease development. However, the analyses reported in this paper were meant to be hypotheses generating and we hope that these preliminary findings will stimulate further studies. Concomitant use of statins did not confound our main findings notably.

Previous studies have found that AD patients had higher levels of ceramides in the middle frontal cortex [30] and white matter [29]; levels peaked in early mild dementia (CDR = 0.5) [29]. One CSF study reported higher ceramide levels in moderate versus mild or severe AD [48], while another examining brain tissue reported that the gene expression patterns of enzymes participating in the sphingolipid metabolism pathway varied by AD severity [49]. Elevated SM levels were found in the inferior parietal lobe of AD patients, and had a strong positive correlation with the number of amyloid-beta plaques [50]. In contrast, one study reported lower SM levels in the middle frontal gyrus of AD patients [30]. The conflicting results could be due to the stage of the disease process studied. A recent study with human plasma has found that serum SM and ceramides varied by time to onset of memory impairment, i.e., the levels were higher pre-symptomatically but lower at the time of impairment but also that higher ceramide levels in prodromal AD was associated with greater hippocampal atrophy [33]. Our findings are consistent with those reported previously in the literature, but also extend them by providing a deeper and broader characterization at the molecular species levels and providing correlations with dementia severity and genotype.

While the present results are intriguing, the exact biochemical mechanism(s) leading to the reduction of these peripheral blood lipids in people with memory loss still remains unknown. However, investigators have speculated that changes in blood components may be representative of structural and metabolic changes in the brain [33], [51]. SM species are important cellular membrane constituents which are tightly associated with cholesterol in construction, metabolism and transport, and which are enriched in lipid rafts. SM species are metabolized into ceramides, an important second messenger involving in regulation of cellular differentiation, proliferation, and apoptosis [52]. Ceramides are also intermediates linking inflammatory cytokines to insulin resistance [53] and subclinical atherosclerosis [54], [55]; all of which are associated with AD pathogenesis.

**Figure 10:**
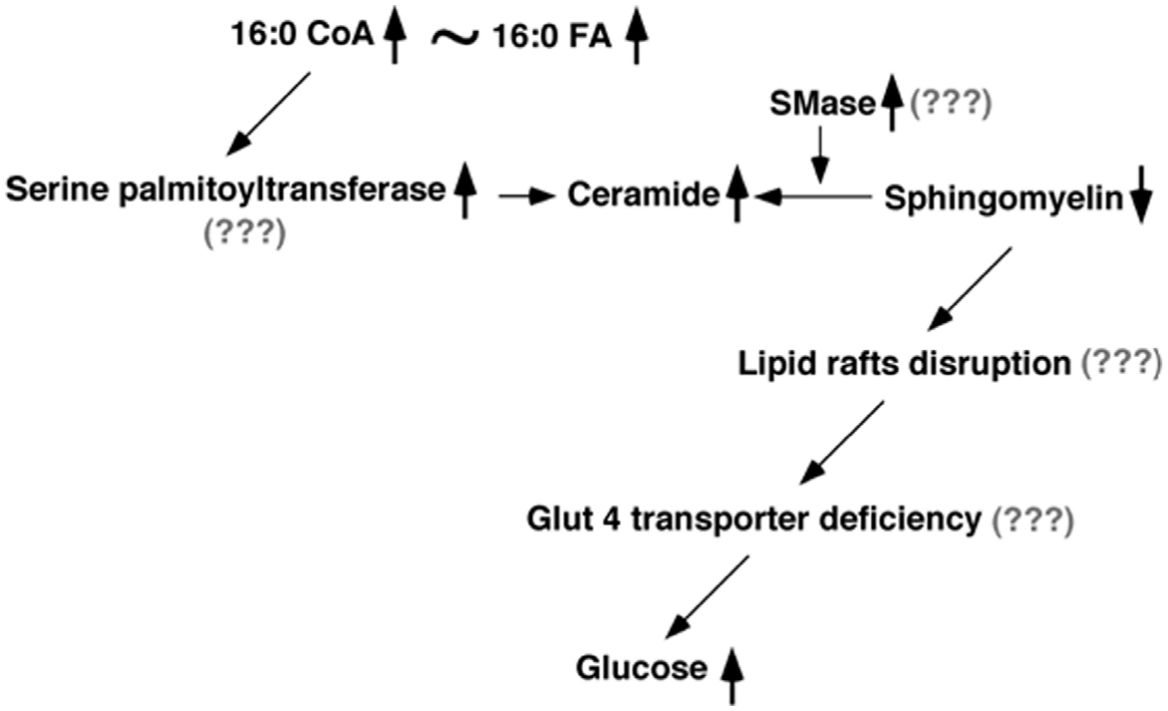
A proposed working model of the altered sphingolipid metabolism in Alzheimer's disease. See text for details.

To this end, we propose a neurochemical working model based on our results (Figure 10). In the model, the increased ceramide mass levels might result either from the accelerated SM hydrolysis, which would involve either the increased levels or activities of sphingomyelinase (SMase) or increased biosynthesis, which would be largely associated with serine palmitoyltransferase. The former is consistent with the lower mass levels of SM species as determined in the current study. The latter is supported by our metabolomic studies that saturated fatty acids and their derivatives (e.g., monoacylglycerol) accumulate in CSF (unpublished data). SM deficiency could lead to alterations in lipid raft formation, thereby changing Glut 4 translocation and leading to glucose accumulation in plasma as previously obtained [56], [57]. This model clearly is speculative and has many unanswered questions that need further testing in subsequent studies.

There are some limitations to the study that need to be considered. First is the issue of type 1 error. Although the sample size of 52 participants is reasonable for a metabolomic analysis, the number of comparisons raises the potential for spurious findings. To control for this issue we restricted our comparisons to focused *a priori* hypotheses of disturbances in lipid metabolism and we employed relatively conservative p values for statistical significance. Other sampling issues such as participation biases in our groups, differences in age, diet, medical status and concomitant medications, as well as genetic differences are also possible but difficult to control within the logistics of recruiting a clinically relevant sample. Regardless, replication of the results from this study is important and should be conducted in more diverse and larger clinical samples followed longitudinally over time. Additionally, this study included patients with diagnosed AD dementia that were in the mild to moderate stages. Future studies would benefit from the inclusion of patients with well-characterized mild cognitive impairment, other dementing disorders as well as asymptomatic patients with preclinical disease – i.e. other positive biomarkers. Further, given the possible links between lipid alterations and amyloidogeneis, it would be of great interest to examine the relationship between lipidomics and amyloid biomarkers, such as CSF or PET scans. It would also be of great interest to analyze CSF and plasma lipidomics in the same subjects to examine their relationships and relative efficacy as biomarkers. Such studies will be essential to fully harness the potential of lipidomics findings into biomarkers.

In summary, using shotgun lipidomics, we demonstrate significant disruptions in the sphingolipidome in plasma obtained from AD patients compared to normal controls. Our results provide new insights into the relationship between lipid biochemistry and neuronal dysfunction in early AD and highlight the promise of peripheral lipidomics for the identification of potential new biomarkers. Deeper analysis of possible perturbations in other lipid classes is underway.
